# Supplementary material for: COZOID: contact zone identifier for visual analysis of protein-protein interactions
Source: BMC Bioinformatics. 2018 Apr 6;19:125. doi: 10.1186/s12859-018-2113-6 (PMC5889581; doi:10.1186/s12859-018-2113-6)
Supplement: Supplementary file 2 — Software build. Executable binary file of the software tool. (ZIP 118,784 kb) [file 12859_2018_2113_MOESM2_ESM.zip › cozoid/java/docs/beansbinding-1.2.1-doc.zip!/org/jdesktop/el/impl/parser/ELParserTreeConstants.html]

ELParserTreeConstants (Beans Binding)


---


|  |  |  |
| --- | --- | --- |
| **Overview** \| **Package** \| **Class** \| **Use** \| **Tree** \| **Deprecated** \| **Index** \| **Help** | |  |
| **PREV CLASS**   **NEXT CLASS** | **FRAMES**    **NO FRAMES**     **All Classes** |  |
| SUMMARY: NESTED \| FIELD \| CONSTR \| METHOD | DETAIL: FIELD \| CONSTR \| METHOD |  |


---


## org.jdesktop.el.impl.parser Interface ELParserTreeConstants

**All Known Implementing Classes:**: ELParser

---

``` public interface ELParserTreeConstants ```

---

| **Field Summary** | |
| --- | --- |
| `static int` | `JJTAND` |
| `static int` | `JJTBRACKETSUFFIX` |
| `static int` | `JJTCHOICE` |
| `static int` | `JJTCOMPOSITEEXPRESSION` |
| `static int` | `JJTDEFERREDEXPRESSION` |
| `static int` | `JJTDIV` |
| `static int` | `JJTDOTSUFFIX` |
| `static int` | `JJTDYNAMICEXPRESSION` |
| `static int` | `JJTEMPTY` |
| `static int` | `JJTEQUAL` |
| `static int` | `JJTFALSE` |
| `static int` | `JJTFLOATINGPOINT` |
| `static int` | `JJTFUNCTION` |
| `static int` | `JJTGREATERTHAN` |
| `static int` | `JJTGREATERTHANEQUAL` |
| `static int` | `JJTIDENTIFIER` |
| `static int` | `JJTINTEGER` |
| `static int` | `JJTLESSTHAN` |
| `static int` | `JJTLESSTHANEQUAL` |
| `static int` | `JJTLITERALEXPRESSION` |
| `static int` | `JJTMINUS` |
| `static int` | `JJTMOD` |
| `static int` | `JJTMULT` |
| `static int` | `JJTNEGATIVE` |
| `static java.lang.String[]` | `jjtNodeName` |
| `static int` | `JJTNOT` |
| `static int` | `JJTNOTEQUAL` |
| `static int` | `JJTNULL` |
| `static int` | `JJTOR` |
| `static int` | `JJTPLUS` |
| `static int` | `JJTSTRING` |
| `static int` | `JJTTRUE` |
| `static int` | `JJTVALUE` |
| `static int` | `JJTVOID` |

**Field Detail**

### JJTCOMPOSITEEXPRESSION

```
static final int JJTCOMPOSITEEXPRESSION
```

**See Also:**: Constant Field Values

---


### JJTLITERALEXPRESSION

```
static final int JJTLITERALEXPRESSION
```

**See Also:**: Constant Field Values

---


### JJTDEFERREDEXPRESSION

```
static final int JJTDEFERREDEXPRESSION
```

**See Also:**: Constant Field Values

---


### JJTDYNAMICEXPRESSION

```
static final int JJTDYNAMICEXPRESSION
```

**See Also:**: Constant Field Values

---


### JJTVOID

```
static final int JJTVOID
```

**See Also:**: Constant Field Values

---


### JJTCHOICE

```
static final int JJTCHOICE
```

**See Also:**: Constant Field Values

---


### JJTOR

```
static final int JJTOR
```

**See Also:**: Constant Field Values

---


### JJTAND

```
static final int JJTAND
```

**See Also:**: Constant Field Values

---


### JJTEQUAL

```
static final int JJTEQUAL
```

**See Also:**: Constant Field Values

---


### JJTNOTEQUAL

```
static final int JJTNOTEQUAL
```

**See Also:**: Constant Field Values

---


### JJTLESSTHAN

```
static final int JJTLESSTHAN
```

**See Also:**: Constant Field Values

---


### JJTGREATERTHAN

```
static final int JJTGREATERTHAN
```

**See Also:**: Constant Field Values

---


### JJTLESSTHANEQUAL

```
static final int JJTLESSTHANEQUAL
```

**See Also:**: Constant Field Values

---


### JJTGREATERTHANEQUAL

```
static final int JJTGREATERTHANEQUAL
```

**See Also:**: Constant Field Values

---


### JJTPLUS

```
static final int JJTPLUS
```

**See Also:**: Constant Field Values

---


### JJTMINUS

```
static final int JJTMINUS
```

**See Also:**: Constant Field Values

---


### JJTMULT

```
static final int JJTMULT
```

**See Also:**: Constant Field Values

---


### JJTDIV

```
static final int JJTDIV
```

**See Also:**: Constant Field Values

---


### JJTMOD

```
static final int JJTMOD
```

**See Also:**: Constant Field Values

---


### JJTNEGATIVE

```
static final int JJTNEGATIVE
```

**See Also:**: Constant Field Values

---


### JJTNOT

```
static final int JJTNOT
```

**See Also:**: Constant Field Values

---


### JJTEMPTY

```
static final int JJTEMPTY
```

**See Also:**: Constant Field Values

---


### JJTVALUE

```
static final int JJTVALUE
```

**See Also:**: Constant Field Values

---


### JJTDOTSUFFIX

```
static final int JJTDOTSUFFIX
```

**See Also:**: Constant Field Values

---


### JJTBRACKETSUFFIX

```
static final int JJTBRACKETSUFFIX
```

**See Also:**: Constant Field Values

---


### JJTIDENTIFIER

```
static final int JJTIDENTIFIER
```

**See Also:**: Constant Field Values

---


### JJTFUNCTION

```
static final int JJTFUNCTION
```

**See Also:**: Constant Field Values

---


### JJTTRUE

```
static final int JJTTRUE
```

**See Also:**: Constant Field Values

---


### JJTFALSE

```
static final int JJTFALSE
```

**See Also:**: Constant Field Values

---


### JJTFLOATINGPOINT

```
static final int JJTFLOATINGPOINT
```

**See Also:**: Constant Field Values

---


### JJTINTEGER

```
static final int JJTINTEGER
```

**See Also:**: Constant Field Values

---


### JJTSTRING

```
static final int JJTSTRING
```

**See Also:**: Constant Field Values

---


### JJTNULL

```
static final int JJTNULL
```

**See Also:**: Constant Field Values

---


### jjtNodeName

```
static final java.lang.String[] jjtNodeName
```


---


|  |  |  |
| --- | --- | --- |
| **Overview** \| **Package** \| **Class** \| **Use** \| **Tree** \| **Deprecated** \| **Index** \| **Help** | |  |
| **PREV CLASS**   **NEXT CLASS** | **FRAMES**    **NO FRAMES**     **All Classes** |  |
| SUMMARY: NESTED \| FIELD \| CONSTR \| METHOD | DETAIL: FIELD \| CONSTR \| METHOD |  |


---
